# Compensated Cirrhosis in a 40-Year-Old Woman With Progressive Familial Intrahepatic Cholestasis Type 3

**DOI:** 10.14309/crj.0000000000002237

**Published:** 2026-07-10

**Authors:** Yee Hui Yeo, Danielle Hutchings, Maha Guindi, Paul Martin

**Affiliations:** 1 Department of Medicine, Karsh Division of Gastroenterology and Hepatology, Cedars-Sinai Medical Center, Los Angeles, CA; 2 Comprehensive Transplant Center, Cedars-Sinai Medical Center, Los Angeles, CA; 3 The Department of Pathology & Laboratory Medicine, Cedars-Sinai Medical Center, Los Angeles, CA

**Keywords:** progressive familial intrahepatic cholestasis type 3, *ABCB4*, heterozygous, missense mutation, compensated cirrhosis

## Abstract

Progressive familial intrahepatic cholestasis type 3 (PFIC3) is caused by mutations in *ABCB4* encoding canalicular phospholipid transporter multidrug resistance protein 3, causing advanced liver disease in childhood or early adulthood. We describe a 40-year-old woman with PFIC3 with a relatively indolent disease course. She first presented in childhood with pruritus, with episodes precipitated by estrogen exposure and pregnancy. Genetic testing confirmed compound heterozygous *ABCB4* variants. She has well-preserved synthetic function with slow progression to cirrhosis at the age of 40 years. Immunohistochemistry demonstrated canalicular multidrug resistance protein 3 staining. This case illustrates the phenotypic heterogeneity of PFIC3 and highlights delayed disease progression.

## INTRODUCTION

Progressive familial intrahepatic cholestasis type 3 (PFIC3) is a rare autosomal recessive liver disease caused by mutations in the adenosine triphosphate-binding cassette subfamily B member 4* (ABCB4)* gene encoding the multidrug resistance protein 3 (MDR3). MDR3 functions as a canalicular phospholipid transporter, facilitating the secretion of phosphatidylcholine into bile and protecting the biliary epithelium from the detergent effects of bile acids.^1^ Defective MDR3 function results in cholestasis with characteristically elevated gamma-glutamyl transferase (GGT) (in contrast to PFIC1 and PFIC2, which have normal GGT).^2^ Histologically, PFIC3 is characterized by portal fibrosis with bile duct proliferation and progression to biliary cirrhosis.^3^ The disease typically presents in late infancy or childhood with overt cholestatic symptoms (pruritus, jaundice), with progression to portal hypertension and end-stage liver disease by the first or second decade of life.^4^ However, a minority of patients have atypically mild or late presentations, and PFIC may not be recognized in the absence of a family history. These milder phenotypes reflect mutations that allow some residual MDR3 function.

Ursodeoxycholic acid (UDCA) is recommended as therapy for PFIC3 and can mitigate symptoms in at least some patients with biochemical improvement. UDCA works by replacing hydrophobic bile acids with a less toxic, hydrophilic bile acid and by upregulating residual MDR3 protein expression on the canalicular membrane.^5^

We describe a 40-year-old woman with PFIC3 with a relatively indolent progression of liver disease over many years.

## CASE REPORT

The patient, an only child, presented with pruritus without jaundice at the age of 10 years and was noted to have hepatomegaly while living in Eastern Europe. Evaluation revealed elevated alkaline phosphatase and transaminases. Liver biopsy was nondiagnostic, and UDCA therapy was initiated. Over the subsequent 3 decades, she experienced intermittent cholestatic flares approximately once yearly with pruritus, occasional jaundice, and elevated liver enzymes. These episodes typically lasted 1–2 weeks and resolved spontaneously with supportive care. Between episodes, she remained completely asymptomatic with normalization of liver tests. The symptoms were exacerbated when she took oral contraceptive pills and isotretinoin (Accutane).

**Figure 1.**
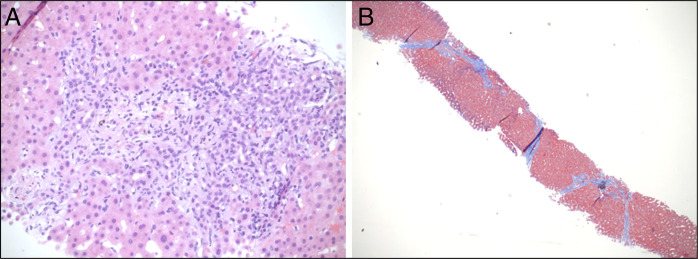
(A) Portal tract demonstrating ductular reaction with mild mixed inflammation. (B) Masson trichrome stain demonstrating portal/periportal fibrosis with focal bridging septa.

She sought medical attention after immigration to the United States, with a liver biopsy at the age of 22 years showing an absence of portal inflammation or fibrosis. A subsequent biopsy at the age of 26 years revealed portal tracts with ductular reaction and portal/periportal fibrosis with focal bridging septa (Figure 1). Endoscopic retrograde cholangiopancreatography was normal.

**Figure 2.**
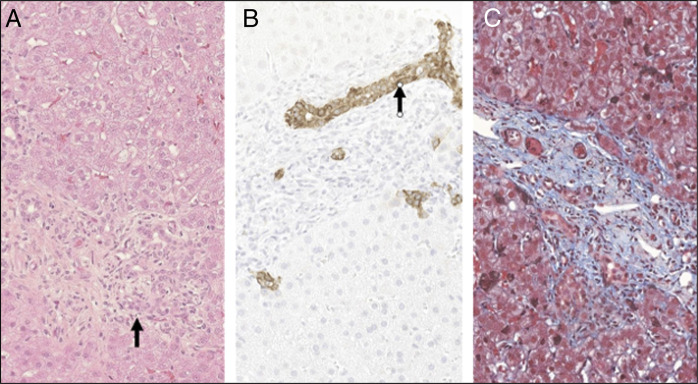
(A, left) Portal tract demonstrating ductular reaction (arrow) with mild inflammation. (B, middle) Keratin 7 immunostain highlighting ductular reaction (arrow). (C, right) Masson trichrome stain demonstrating portal fibrosis.

She next presented at the age of 28 years, at 12 weeks gestation, with pruritus, dark urine, and clay-colored stools. Laboratory testing showed alkaline phosphatase 132 U/L, total bilirubin 2.3 mg/dL (direct 1.5 mg/dL), aspartate aminotransferase 134 U/L, alanine aminotransferase 301 U/L, and total bile acids 277.6 μmol/L. A magnetic resonance cholangiopancreatography demonstrated a normal biliary tree and pancreatic duct. Repeat liver biopsy demonstrated bridging fibrosis and bile duct injury (Figure 2). She underwent genetic testing for PFIC, which revealed a heterozygous p.S320F mutation and a heterozygous p.G722A variant (most likely a deleterious variant according to the SIFT and PolyPhen algorithms). This confirmed a diagnosis of PFIC type 3 due to the *ABCB4* gene mutation. Notably, her GGT levels were normal during this flare. Following delivery, her pruritus partially improved. At postpartum follow-up, liver tests normalized and symptoms resolved. Her son underwent *ABCB4* genetic testing, which was negative.

**Figure 3.**
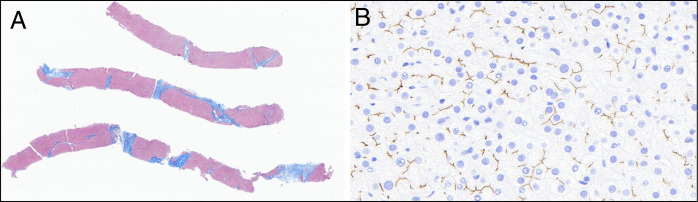
(A) Masson trichrome stain highlights cirrhosis with variably sized and irregularly shaped nodules. (B) Multidrug resistance protein 3 immunohistochemistry shows intact canalicular staining.

Her pruritus continued to follow a relapsing-remitting course, triggered by infections (cold sore in 2013 and respiratory infection in 2025). These flares were self-limited. At the age of 40 years, she presented with mild pruritus, coinciding with recovery from a respiratory illness. Pertinent blood results included alanine aminotransferase of 39 U/L, aspartate aminotransferase of 20 U/L, alkaline phosphatase of 84 U/L, total bilirubin of 0.6 mg/dL, and international normalized ratio of 1.1. Platelets were preserved at 172 × 10^3/μL. Abdominal ultrasound showed nodular liver contour. Liver biopsy was repeated for prognostic purposes. Histopathology revealed cirrhosis with variably sized and irregularly shaped nodules compatible with biliary pattern cirrhosis (Figure 3). Ductular reaction was largely absent. Canalicular staining for MDR3 was preserved on immunohistochemistry, suggesting residual MDR3 expression. No copper, iron overload, or features of autoimmune liver disease were seen.

The patient remains well-compensated, asymptomatic, and continues UDCA therapy. She is monitored with semiannual hepatocellular carcinoma surveillance, periodic laboratory assessments, and ongoing evaluation for portal hypertension. Bone health is addressed with vitamin D supplementation and interval dual-energy X-ray absorptiometry scans, which have thus far remained normal.

## DISCUSSION

This case illustrates an atypically mild, slowly progressive course of PFIC type 3, likely attributable to partial MDR3 function. PFIC3 is generally progressive, with cirrhosis and portal hypertension developing in childhood or adolescence.^4^ Our patient, by contrast, has remained largely asymptomatic into her fourth decade, with normal laboratory liver function despite underlying cirrhosis.

Adult presentation of this disorder has been recognized in subjects with heterozygous or milder *ABCB4* mutations that permit some MDR3 activity.^6^ In PFIC3, MDR3 immunohistochemistry can be variable, with some patients demonstrating absent staining and others showing preserved canalicular expression despite impaired function.^7,8^ Therefore, a positive MDR3 stain does not exclude PFIC3; genetic testing remains the definitive diagnostic tool. The patient carried a heterozygous p.S320F missense mutation and a heterozygous p.G722A variant, potentially deleterious variant based on in silico prediction tools. However, the preserved canalicular MDR3 staining suggests residual protein expression despite impaired function.

The prolonged clinical course in our patient is also notable when compared with previously reported adult presentations of PFIC3. A previous case reported that heterozygous ABCB4 mutations developed cirrhosis in adulthood and improved with UDCA.^6^ Similarly, our patient exhibited a relatively mild phenotype despite progressive fibrosis. However, our case is distinguished by a history of hormonally triggered cholestatic episodes.

Pregnancy-associated cholestasis ultimately led to the diagnosis. The patient's pruritus was essentially an episode of intrahepatic cholestasis of pregnancy (ICP) with elevated bile salts. Heterozygous or mild mutations in *ABCB4 *are risk factors of ICP, since the high reproductive hormonal levels may be associated with impaired transportation of bile salt.^9,10^ This is consistent with our patient's flare when taking oral contraceptive pills during early adulthood, as noted in other patients^11^ Many women with *ABCB4* mutations present first with ICP and are later found to have chronic cholestatic liver disease.^9^ This patient's pregnancy-associated cholestasis ultimately led to the diagnosis of PFIC3 after years of otherwise unexplained cholestatic episodes. Together with her prior intolerance of oral contraceptive therapy, this observation supports the role of hormonal triggers in unmasking *ABCB4*-related disease and highlights the importance of considering genetic cholestatic disorders in women presenting with severe gestational pruritus.

## DISCLOSURES

Author contributions: Patient recruitment: YH Yeo and P. Martin. Conception and design: P. Martin. Collection and assembly of data: all authors. Manuscript drafting: YH Yeo. Critical review of the manuscript: All authors. P. Martin is the article guarantor.

Declaration of generative AI and AI-assisted technologies in the manuscript preparation process: During the preparation of this work the author used GPT-5.2 in order to edit the content's grammar and language. After using this tool/service, the author reviewed and edited the content as needed and takes full responsibility for the content of the published article.

Financial disclosure: None to report.

Informed consent was obtained for this case report.

## ABBREVIATIONS:

ABCB4: adenosine triphosphate-binding cassette subfamily B member 4
MDR3: multidrug resistance protein 3
PFIC3: progressive intrafamilial intrahepatic cholestasis type 3
